# Melatonin Attenuates the Progression of Osteoarthritis in Rats by Inhibiting Inflammation and Related Oxidative Stress on the Surface of Knee Cartilage

**DOI:** 10.1111/os.13408

**Published:** 2022-07-27

**Authors:** Chenghui Ke, Hongyun Li, Dan Yang, Hao Ying, Hongwen Zhu, Jian Wang, Jun Xu, Lin Wang

**Affiliations:** ^1^ Department of Orthopaedics, Shanghai Children's Hospital, School of Medicine Shanghai Jiao Tong University Shanghai China; ^2^ Department of Anesthesiology, Shanghai Children's Hospital, School of Medicine Shanghai Jiao Tong University Shanghai China; ^3^ Tianjin Hospital, Tianjin Academy of Integrative Medicine Tianjin China; ^4^ Tongji University School of Medicine Shanghai China

**Keywords:** Inflammatory factor, Melatonin antagonist, Osteoarthritis model, Papain, SD male rats

## Abstract

**Objective:**

To investigate the correlation between melatonin and osteoarthritis (OA) in rats. To explore the relevant mechanisms in the occurrence and development of osteoarthritis in rats, and to further understand the disease of osteoarthritis.

**Methods:**

Forty healthy 6‐month‐old male SD rats were randomly divided into two groups: sham and drug intervention groups. Pre‐OA modeling, enzyme‐linked immunosorbent assay was employed to detect the levels of IL‐1β, IL‐6, COX‐2, and melatonin in the serum of the rats in each group. For OA modeling, we administered an injection of papain into the knee cavity of all rats. The levels of IL‐1β, IL‐6, and COX‐2 in the serum of rats in each group were detected 2 weeks after the modeling. Additionally, 2 weeks after the modeling, the rats in the drug intervention group were intraperitoneally injected with melatonin antagonists. The rats in the sham group were intraperitoneally injected with normal saline for 2 weeks. The levels of IL‐1β, IL‐6, and COX‐2 in the serum of each group were measured at the second, third, and fourth weeks after the drug intervention, and the levels of melatonin in the serum were measured at the second week after the drug intervention. Finally, the rats were euthanized by cervical dislocation, and pathological sections were collected from the knee joint to observe the pathological tissue changes under a microscope, and Mankin score was determined. The independent samples *t*‐test method was used for analysis.

**Results:**

The imaging examination after the drug intervention showed that the modeling of knee osteoarthritis in rats was successful. In the pathological findings, HE staining showed a legible cartilage structure of each layer, with cartilage proliferation and partial cartilage tearing to the radial layer. The tide line was intact; toluidine blue staining revealed more obvious changes. The differences among the mean values of IL‐6, IL‐1β, and COX‐2 measured in each period were statistically significant (*t* = 5.50, *p* < 0.05). The measured mean values of IL‐6, IL‐1β, and COX‐2 revealed statistically significant differences among the groups (*t* = 2.01, *p* < 0.05). The intergroup comparison of the Mankin scores in each period showed statistically significant differences.

**Conclusion:**

Melatonin may inhibit inflammation and associated oxidative stress on the surface of knee cartilage. It may be related to the repair and regeneration of articular surface cartilage during the development of OA in the rat knee joint.

## Graphical abstract

In this experiment, after the above three pro‐inflammatory factors were modeled in osteoarthritis (OA), their blood content levels gradually increased with the progress of the experiment, and they were statistically significant, indicating that in the case of melatonin reduction, each of the index increased significantly, indicating that the mechanism of melatonin in the repair and regeneration of cartilage on the knee joint may be caused by inhibiting inflammation and related oxidative stress on the surface of knee cartilage. We preliminarily explored the correlation between melatonin and OA, and even the correlation between melatonin and IL‐1β, IL‐6 and COX‐2, and clarified the effect of melatonin on OA mechanism. However, there are still some shortcomings in this experiment, and the correlation between melatonin and OA at the molecular level or even the gene level needs to be further explored to provide a basis for clinical prevention and treatment of OA.

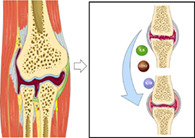

## Introduction

Osteoarthritis (OA) is an orthopaedic disease that often occurs in the elderly population, with clinical manifestations of joint swelling and pain, functional impairment, and pathological features of progressive destruction of the articular cartilage and degenerative changes in the subchondral bone, periarticular ligaments, and synovial tissue. The prevalence of this chronic disease and most common chronic arthropathy caused by a number of pathogenic factors, increases with age and predominantly affects the elderly over 65 years.^1^ The knee, hand, hip, and spine are the most commonly injured joints in OA, with manifestations of joint pain and limited activities. OA has become an important cause of disability in the elderly.^2^, ^3^ Although several risk factors associated with OA, such as genetic predisposition, aging, obesity, and joint misalignment, have been found, the pathogenesis of OA remains unclear.^4^, ^5^ At present, most scholars believe that osteoarthritis is related to the repair and regeneration of articular surface cartilage.^6^ Therefore, OA‐related studies aimed at the retardation of OA progression, or even its reversal, are of critical significance.

Animal models are the main way to elucidate the occurrence and development mechanism of knee osteoarthritis and establish novel methods for its treatment. Rats are widely used as experimental animals in animal OA models because of their accessibility, relatively low cost, short modeling time, and high survivability. Current modeling methods for OA include intra articular injection, spontaneous animal models, and surgical modeling.^7^, ^8^ The most commonly employed modeling method is the intra‐articular injection of papain due to its shorter induction effect and higher success rates than those of other drugs^9^; it can reduce the chance of infection and death in animals. It is a mature and reliable modeling method with a high success rate and a large number of precedents reported in previous modeling studies. Therefore, in this experiment, papain was injected into the knee joint cavity to establish an OA model. Studies have shown a correlation between melatonin and osteoarthritis, but the underlying mechanisms of interaction need to be further investigated. In this experiment, the correlation between melatonin and OA, especially the correlation between melatonin andInterleukin‐6 (IL‐6), Interleukin‐1β (IL‐1β), and Cyclooxygenase‐2 (COX‐2) in OA development, was investigated through the detection of OA‐related inflammatory indexes and pathological observation.

The research objectives of this experiment were as follows: (i) To study the possible correlation between the artificial control of the content of melatonin in rats and the progression of knee osteoarthritis, and to further explore the results of its imaging, pathology, and related inflammation indicators. Possible factors for the progression of osteoarthritis in rats were also examined; (ii) To analyze the correlation between melatonin and the results of related imaging, pathology, and inflammation indicators, explore the possible correlation between related inflammation indicators and osteoarthritis in rats, and understand the mechanism of the influence of melanin on osteoarthritis progression in rats; (iii) To explore the correlation between related inflammatory indexes and osteoarthritis in rats, as well as to evaluate the possible influencing factors of the clinical progression of osteoarthritis in rats.

## Methods

In our study, we used 40 healthy 6‐month‐old male SD rats (clean grade) weighing 200–250 g. All animals were purchased from Minhou County Wu Experimental Animal Trade Co., Ltd. (Fuzhou, China). All rats were adaptively fed for 1 week before the experiment. They were housed indoors in group cages at a controlled room temperature of (22 ± 2)°C, with natural light or lights on from 8:00 to 20:00, and lights off for the rest of the day. The rats were fed by a specially assigned person and drank water *ad libitum*. The feed and the height of the drinking water were adjusted according to their growth rate. All feed was purchased from Minhou County Wu Experimental Animal Trade Co., Ltd. All animal experiments were performed according to protocols approved by the Ethical Committee of Experimental Animal Center affiliated to Medicine School of Tongji University. The approval No. is TJLAC‐014‐012.

All 40 healthy 6‐month‐old SD female rats were randomly divided into a sham group and a melatonin antagonist (MTX) injection group, 20 rats per group, and were fed adaptively for 1 week. After the rats in each group were successfully anesthetized with 10% chloral hydrate (300 mg/kg) intraperitoneally pre‐OA modeling, X‐ray and MRI were performed, and blood was collected from the severed tails in a prone position for the determination of the studied indexes.

### OA Modeling: Procedures and Drug Intervention

After 1‐week adaptive feeding, the rats in both groups underwent MRI of the right knee joint and whole‐body X‐ray pre‐OA modeling. Then, each of the rats in both groups was given an intra‐articular injection of papain at a concentration of 4%, 1 ml/kg/time on days 1, 4, and 7, which was a total number of three injections in the right knee joint. The rats were fed by a specially assigned person after each dose and were free to drink and eat after the operation. Next, the animals were fed for another 2 weeks after drug administration. Two weeks later, rats were randomly selected from each group for MRI photography of the knee joint, and blood was drawn from the severed tail to detect the relevant blood indexes to determine the successful preparation of the OA animal model.

Further, rats in each group were tail‐clamped to draw blood at 2, 3, 4, and 5 weeks after modeling, and blood indicators (IL‐1β, IL‐6, and COX‐2) were detected. MRI was performed in the 5th week. Then, the rats in each group were sacrificed by cervical dislocation, and pathological tissue specimens were collected from of each group. Glass slides were made to observe the knee joints of the rats and examine any arthritis‐related changes. The Mankin score was employed for the assessment of the pathological results. Mankin scoring system is a sensitive method and provides an examination of even slight degenerative changes in articular cartilage. Features of cell, change in matrix structure, tidemark zone, formation of pannus, the characteristic of articuler surface, in degenerative cartilage can be evaluated by this scoring system. It is an adequate system for comprising of all morphological changes seen in OA and having little difference between intra‐and interobserver reliability. It was found that the histopathological gradation of the severity of OA according to Mankin appeared to be directly correlated with the metabolic state of the chondrocytes in the different stages of OA.

## Pathological Tissue of Rat Knee Joint

All rats were sacrificed by cervical dislocation. The knee joints of both sides of the rats were surgically excised under sterile conditions. The pathological specimens of the surface cartilage of all rat knee joints were taken, decalcified in a specific liquid, and made into pathological sections. HE staining was used to observe the tissue structure and cell morphology of rat knee joints, and the morphological differences between normal and diseased knee joints of rats were compared; toluidine blue staining was used to observe cartilage degeneration, chondrocyte necrosis and calcification, and cartilage degeneration on the surface of rat knee joints. Matrix fissures, collagen microscopic hyperplasia and other pathological changes. The surface cartilage of the rat knee joint was observed under the electron microscope at low magnification (40 × 10) to identify and judge the progression of rat knee arthritis.

### Statistical Analysis

SPSS 17.0(SPSS Inc. USA) was used to analyze the data, and the measurement data were expressed as mean ± SD. Descriptive statistics analyses were conducted of the differences between IL‐1β, IL‐6, COX‐2, whereas the Mankin scores and a *t*‐test were implemented for the comparisons of IL‐1β, IL‐6, and COX‐2. *p* < 0.05 was considered to indicate statistically significant differences.

## Results

### Imaging Results

**Fig. 1:**
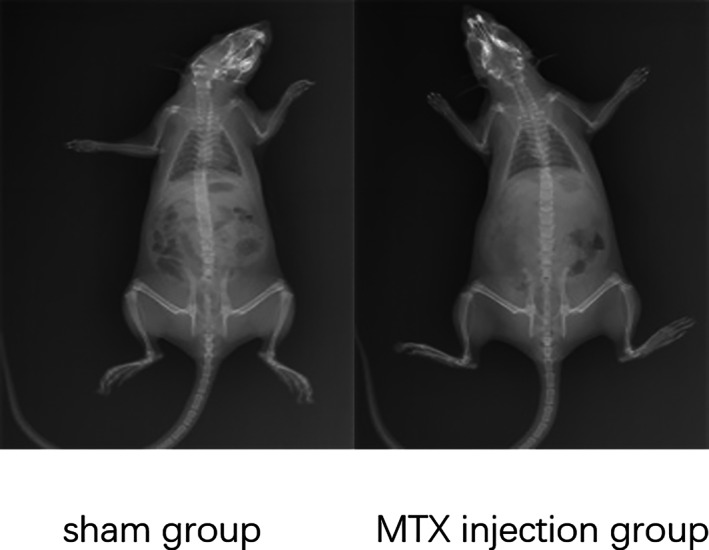
X‐ray of rats in the sham and melatonin antagonist injection groups before surgery. The whole‐body skeleton of rats in each group was normal before surgery, and no bilateral knee lesions were found in any of the rats, which met the experimental requirements. The parameters were as follows: 41 kV and 2.80 mAs

**Fig. 2:**
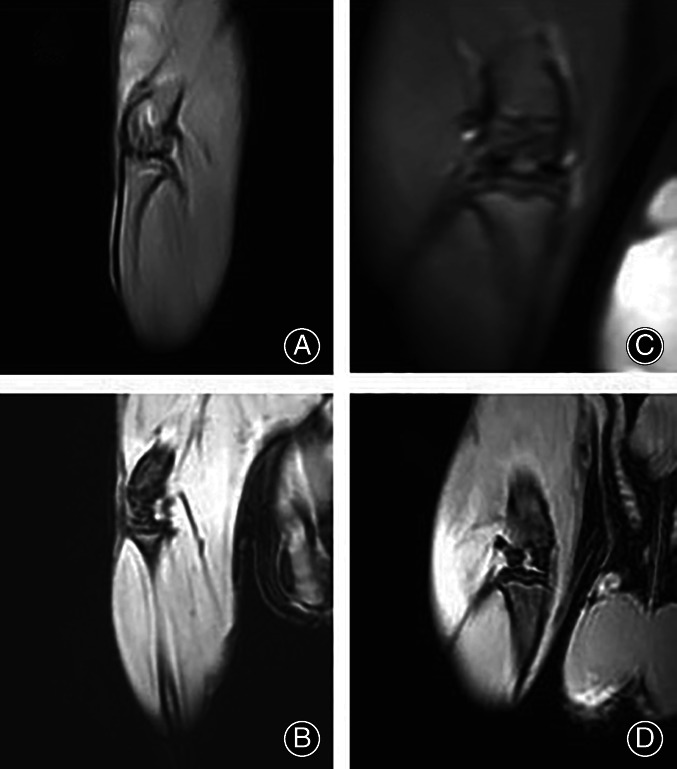
In the first week of the experiment, MRI of rats before modeling: (A, B) MRI results of the knee joints of rats before modeling; 4 weeks after the beginning of the experiment (C and D) after modeling of knee osteoarthritis of rats MRI results of rat knee joint. Compared with the MRI of the rat knee joint before modeling, it can be seen that the cartilage on the surface of the knee joint was gross; the signal was discontinuous at some levels. The overall structure of the knee joint was intact, with no obvious wear and tear and osteophyte in the subchondral bone

The whole‐body skeleton of the rats in each group was normal before surgery. No bilateral knee lesions were found in any of the rats, which met the experimental requirements (Figure 1). Figure 2A,B is the images of PDW and 3D‐Watsc sequences of the right knee joint of the rats before modeling, which revealed that the structure of the knee joint and the cartilage structure of the joint surface of rats were intact, with continuous signal, smooth surface, and no wear and tear and osteophyte of the subchondral bone; Figure 2C,D is coronal PDW, 3D‐Watsc sequence images after modeling. The cartilage on the surface of the knee joint was gross, and the signal was discontinuous at some levels (Figure 2). The overall structure of the knee joint was intact, with no obvious wear and tear and osteophyte in the subchondral bone.

### Melatonin Antagonist Causes a Severe Loss of Cartilage Matrix

**Fig. 3:**
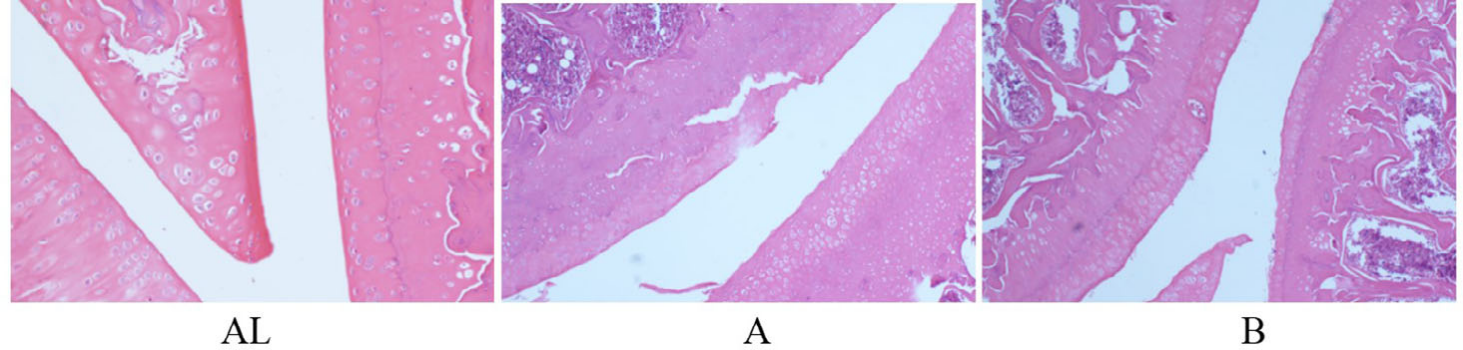
On the 9th week after the start of the experiment, the pathological tissues of the rats in each group after knee osteoarthritis modeling (low power lens: 40 × 10; HE staining). (AL) Left knee joint of the sham group; (A) Right knee joint in the sham group; (B) Right knee joint in the drug intervention group.

In the HE staining results, Figure 3 AL shows the left knee joint of the sham group where the four layers of articular cartilage (shallow layer, transition layer, radiation layer, and calcified layer) were clearly visible, and the tide line was clear and intact. The cartilage surface was smooth, with blue nuclei and uniformly red‐stained cytoplasm. Figure 3A shows that the right knee joint in the sham group where the layers of cartilage structures were legible, while the cartilage surface was not smooth with partial tearing to the radial layer, and tide lines are clear and obvious. Figure 3B illustrates the right knee joint in the drug intervention group, where the cartilage structure of each layer can be identified, with cartilage proliferation and partial cartilage tearing to the radial layer, and the tide line was intact (Figure 3).

**Fig. 4:**
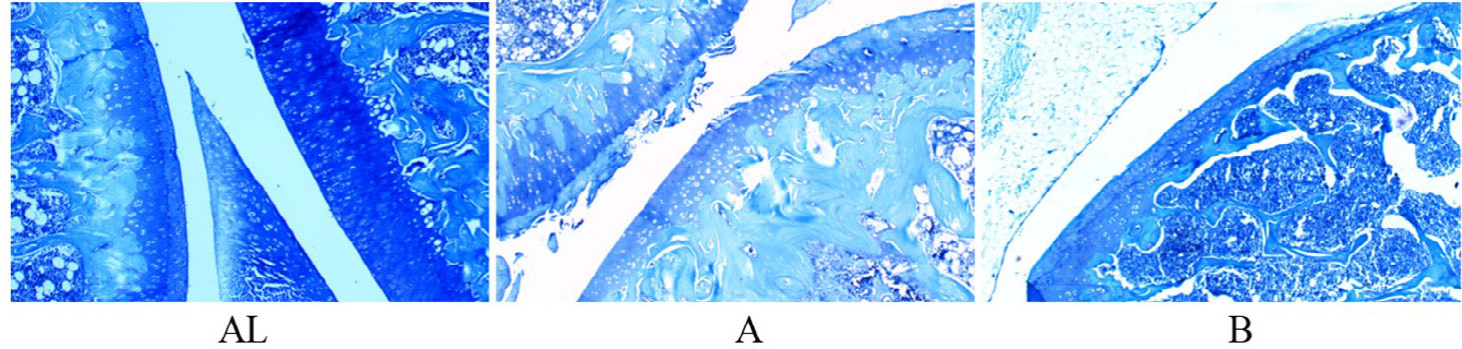
On the 9th week after the start of the experiment, the pathological tissues of the rats in each group after knee osteoarthritis modeling (low power lens: 40 × 10; toluidine blue staining). (AL) Left knee joint of the mice in the sham group; (A) Right knee joint of the rats in the sham group with a mild loss of cartilage matrix staining; (B) Drug intervention group with a moderate to a severe loss of cartilage matrix staining.

In the toluidine blue staining results, Figure 4 AL displays the left knee joint of the mice in the sham group, in which the cartilage matrix was light blue‐purple under toluidine blue staining, the nucleus of chondrocytes was blue, the cytoplasm was not colored, and the cartilage structure was clear. Figure 4A depicts the right knee joint of the rats in the sham group with mild loss of cartilage matrix staining. Figure 4B shows the drug intervention group with moderate to severe loss of cartilage matrix staining (Figure 4).

### Melatonin Inhibits Pro‐Inflammatory Cytokine Formation

As visible in Tables 1, 2, and 3, statistically significant differences were present in the results of the comparison of the mean values of IL‐6, IL‐1β, and COX‐2 measured in each period within each of the groups (*t* = 5.50, *p* = 0.0012). The mean values of IL‐6, IL‐1β, and COX‐2 in each period compared among groups showed a gradual increase as the melatonin levels decreased with statistically significant differences in the results (*t* = 2.01, *p* = 0.001), confirming the involvement of melatonin in the production of pro‐inflammatory cytokines, mainly by inhibiting their formation.

**TABLE 1:** IL‐6 of rats in each group before and after OA modeling (*n* = 20, *x* ± *s*, pg/ml)

Groups	Before OA modeling	2W after OA modeling	2W after drug intervention	3W after drug intervention	4W after drug intervention
Sham group	438.8 ± 130.1	650.3 ± 124.1	828.2 ± 117.1	1047.1 ± 140.1	1255.6 ± 142.5
Drug intervention group	430.7 ± 121.8	724.8 ± 100.1	944.0 ± 106.1	1158.1 ± 86.1	1391.6 ± 100.7
*t* value	2.01	5.26	69.51	7.41	6.55
*p* value	0.0012	0.004	0.058	0.003	0.002

*Note*: For the between‐group mean comparison, indicating the results are statistically significant; for the within‐group mean comparison between “before OA modeling group” and “2W after OA modeling group”, indicating the results are statistically significant; For the mean comparison between “2W after drug intervention group” and “3W after drug intervention group”, indicating the results are statistically significant；For the mean comparison between “3W after drug intervention group” and “4W after drug intervention group”, indicating the results are statistically significant.

**TABLE 2:** IL‐1β of rats in each group before and after OA modeling (*n* = 20, *x* ± *s*, pg/ml)

Groups	Before OA modeling	2W after OA modeling	2W after drug intervention	3W after drug intervention	4W after drug intervention
Sham group	167.2 ± 43.2	219.4 ± 24.1	264.4 ± 42.9	308.2 ± 44.7	357.2 ± 45.3
Drug intervention group	169.3 ± 42.2	277.2 ± 43.1	342.1 ± 106.1	392.1 ± 106.1	460.2 ± 106.1
*t* value	4.72	8.39	38.54	7.59	4.96
*p* value	0.002	0.001	0.058	0.0031	0.0016

*Note*: For the between‐group mean comparison, indicating the results are statistically significant; For the within‐group mean comparison between “before OA modeling group” and “2W after OA modeling”, indicating the results are statistically significant; For the mean comparison between “2W after drug intervention group” and “3W after drug intervention group”, indicating the results are statistically significant；For the mean comparison between “3W after drug intervention group” and “4W after drug intervention group”, indicating the results are statistically significant.

**TABLE 3:** COX‐2 of rats in each group before and after OA modeling (*n* = 20, *x* ± *s*, U/L)

Groups	Before OA modeling	2W after OA modeling	2W after drug intervention	3W after drug intervention	4W after drug intervention
Sham group	173.4 ± 12.6	213.3 ± 24.1	254.7 ± 31.1	296.3 ± 31.0	335.9 ± 32.3
Drug intervention group	198.6 ± 20.6	264.6 ± 22.1	334.4 ± 106.1	403.8 ± 106.1	474.6 ± 106.1
*t* value	9.77	7.22	49.77	8.51	3.96
*p* value	0.002	0.0013	0.058	0.0018	0.0011

*Note*: For the between‐group mean comparison, indicating the results are statistically significant; For the within‐group mean comparison between before OA modeling group and 2W after OA modeling, indicating the results are statistically significant; For the mean comparison between 2W after drug intervention group and 3W after drug intervention group, indicating the results are statistically significant; For the mean comparison between 3W after drug intervention group and 4W after drug intervention group, indicating the results are statistically significant.

The IL‐6 values of the rats in each of the groups before and post‐OA modeling are presented in Table 1. With the progress of the experiment, IL‐6 gradually increased and was significantly correlated to the progression of osteoarthritis in the experimental and control groups of rats.

The IL‐1βvalues of the rats in each of the groups before and post‐OA modeling are presented in Table 2. With the progress of the experiment, IL‐1β gradually increased and was significantly correlated to the progression of osteoarthritis in the experimental and control groups of rats.

The COX‐2 values of the rats in each of the groups before and post‐OA modeling are presented in Table 3. With the progress of the experiment, COX‐2 gradually increased and was significantly correlated to the progression of osteoarthritis in the experimental and control groups of rats.

### Mankin Score

The Mankin score is an adequate histopathological tool that is frequently used for the histopathological classification of the severity of osteoarthritic lesions of the cartilage. A constraint on the validity of this scoring system is the consistency with which cartilage lesions are classified. The intra‐ and interobserver agreement of the 14‐point Mankin score in this study was determined to be adequate. Between observers, 95% of differences were less than −7 points. By a stricter definition of the elements of the Mankin score, the intraobserver differences were reduced only for some observers. On the other hand, the interobserver differences were only slightly lower: between observers 95% of the differences had less than −6 points. The results of Mankin score in each period, and in the comparison of the mean values between groups at the same period, “sham group” and “drug intervention group”, *t* = 7.22, *p* = 0.001, with statistical significance. Therefore, as the experiment proceeded, the degree of OA in each group gradually progressed, and the rats in the melatonin antagonist injection group had more severe symptoms than those in the sham group.

Melatonin of each group was measured at different periods before and post‐OA modeling. In the comparison between‐groups, “sham group” and “drug intervention group”, *t* = 5.84, *p* = 0.0012, indicating the results are statistically significant (Table 4).

**TABLE 4:** Melatonin of each group at different periods before and after OA modeling

Groups	Before OA modeling	2W after drug intervention
Sham group	221.62 ± 26.48	268.20 ± 23.87
Drug intervention group	268.20 ± 23.87	117.48 ± 29.07
*t* value	7.22	5.84
*p* value	0.0012	0.001

*Note*: For the between‐group mean comparison at the same period, indicating the results are statistically significant; for the within‐group mean comparison, indicating the results are statistically significant.

## Discussion

Melatonin may inhibit inflammation and associated oxidative stress on the surface of knee cartilage. It may be related to the repair and regeneration of articular surface cartilage during the development of OA in the rat knee joint.

### Possible Correlation between Melatonin and Knee Osteoarthritis Progression in Rats

Osteoarthritis was initially considered a typical degenerative lesion due to aging, but the understanding of its pathogenesis has changed significantly over the last decade. It is now widely accepted that low levels of inflammation play a crucial role in the development of OA, especially in the early stages.^10^ Melatonin, an indole hormone synthesized and secreted mainly by the pineal body, has been found to be involved in a variety of important physiological processes in the human body, such as sleep, anti‐inflammatory, antioxidant, anti‐aging, and anti‐tumor processes, as well as immune response and reproduction. Mortezaee *et al*.^11^ established that NF‐κB is a ubiquitous transcription factor of oxide that plays an important role in cell survival, cell immunity, and inflammatory responses. Melatonin protects cells from destructive oxidative stress reactions by regulating the NF‐κB signaling pathway and inhibiting the expression of inflammatory factors, such as IL‐1β, IL‐6, and COX‐2, and exerting antioxidant effects.^12^ Bahrampour *et al*.^13^ showed that an elevated oxidative stress reaction in the inflammation microenvironment in injured or diseased joints impeded the differentiation function of bone marrow mesenchymal stem cells (BMSCs), leading to the degradation of the new cartilage. In another study, Liu *et al*.^14^ evidenced that melatonin successfully restored the inhibitory effects of IL‐1β and TNF‐α on cartilage formation in MSCs, which may be related to activities of melatonin, including free radicals scavenging, inhibition of the levels of matrix metalloproteinases (MMPs), and maintenance of superoxide dismutase (SOD) activity. The results of these studies suggest that melatonin regulates the intra‐articular environment and reduces articular cartilage damage *via* its antioxidant, anti‐inflammatory, and anti‐aging activities. Moreover, melatonin has been indicated to induce the differentiation of BMSCs to chondrocytes and to promote chondrocyte repair and regeneration. In this experiment, papain enzyme was injected into the joint cavity to replicate the KOA (knee osseous arthrophlogosis) model, and the correlation between papain enzyme activities and OA was investigated by determination of the inhibition of melatonin levels in rats.

### Melatonin Affects the Results of Related Imaging, Pathology, and Inflammation Indicators

In the imaging examination of this experiment, using partial MRI we found that after modeling, the cartilage on the surface of the knee joint of the examined rats was gross, and the signal was discontinuous. The overall structure of the knee joint was intact, with no obvious wear and tear or osteophyma of the subchondral bone. These results indicated that melatonin was involved mainly in the repair and regeneration of the articular cartilage surfaces during the development of OA.

Moroni *et al*.^15^ investigated the expression of melatonin receptors in human BMSCs, which are the target cells for melatonin target cells for melatonin, as they have a higher ability to differentiate towards cartilage stem cells than such of other tissues.^16^ Yea *et al*.^17^ evidenced that TGF‐β1 induces the differentiation of BMSCs into chondrocytes to further repair articular cartilage damage. Jin *et al*. confirmed that TGF‐β1 was overexpressed in melatonin‐treated chondrocytes. In blood serum culture‐medium, melatonin was able to promote the synthesis of articular cartilage, very possibly through the TGF‐β1 signal transduction pathway.^18^ Additionally, through the expression of HGF (hepatocyte growth factor) and FGF (fibroblast growth factor) in the presence of melatonin, BMSCs was increased.^19^ Wang *et al*.^20^ concluded that FGF stimulates BMSCs to aggregate towards articular cartilage breakage, reconstitutes the fiber substrate, and induces cartilage growth to repair articular cartilage defects. Kremen *et al*.^21^ showed that chondrocytes are the target cells for melatonin target cells for melatonin, and thus melatonin may regulate bone growth and development through the MTl and MT2 receptors in chondrocytes. In the knee joint pathological specimens examined in this experiment, the superficial, transitional, deep, and calcified layers of the articular cartilage were clearly visible. The cartilage surface was not smooth and was partially torn to the deep layer with local chondrocyte hyperplasia. These findings indicated that melatonin is involved mainly in the repair and regeneration of the cartilage during the development of OA. The mechanism of action is the inhibitory effect it exerts on inflammation and the associated oxidative stress reaction on the cartilage surface of the knee joint, mainly deduced from the results of the relevant proinflammatory factors detected in this experiment.

### Correlation between Related Inflammation Indicators and Osteoarthritis in Rats

The inflammatory factor IL‐1β acts as an early degradation factor of articular cartilage and contributes to the development of OA by inducing MMP inhibitor‐1 and MMP imbalance (stimulation of MMP synthesis and inhibition of MMP inhibitor‐1 expression), which further contributes to cartilage breakdown and proteoglycan synthesis inhibition.^22^ Moreover, relevant clinical studies have shown that the level of IL‐1β in the knee joint fluid of OA patients is positively correlated with the severity of the disease.^23^ In this respect, Eriksen *et al*.^24^ found that at the genetic level, microRNA‐193b‐3p regulated the IL‐1β‐reduced expression of MMP 19 in human chondrocytes, suggesting that IL‐1β can promote OA progression. The overproduction of IL‐6 is associated with the pathogenesis of OA.^25^ For example, Stannus *et al*.^26^ found an association between IL‐6 levels and cartilage loss in the knee joints of the elderly and that IL‐6 was upregulated as a proinflammatory cytokine in many inflammatory diseases. Besides, in the clinical research of Liang *et al*.^27^ the expression of IL‐6 in the blood of OA patients was high, but was significantly lower after their cure, and this is another piece of evidence. COX‐2 is an isomeride of cyclooxygenase (COX). MMPs are involved in mediating the collagen degradation process associated with OA, while arachidonic acid metabolites are involved in the mediation of pain and inflammation. COX metabolizes arachidonic acid to form prostaglandin E (PGE), which is then metabolized by prostaglandin E (PGE) synthase to prostaglandin E2 (PGE2), forming a major inflammatory mediator.^28^ Ke *et al*.^29^ revealed that COX‐2 was related to the degradation of the cartilage. In the present experiment, the post‐OA modeling level of the aforementioned three pro‐inflammatory factors in the blood of rats in the drug intervention group gradually increased with statistical significance, indicating that melatonin reduction, significantly increases the values of each of the indexes examined. Thus, the action mechanism of melatonin in the repair and regeneration of the cartilage on the knee joint surface may be implied to be the inhibition of inflammation and the related anti‐oxidative stress reaction.

### Limitations

In this study, a rat model of osteoarthritis was established by papain. Later, by adjusting the physiological secretion of melatonin in rats, the correlation between melatonin and rat osteoarthritis was non‐invasively explored for the first time, which was more in line with rat osteoarthritis the reality of development. In this study, the progression of osteoarthritis in rats was observed through histology, imaging, and blood tests, and the rat arthritis was evaluated by using the relevant scoring standards that are very practical at home and abroad. This study plans to expand the number of studies in rats, further improve the experimental steps, and explore the mechanism of melatonin affecting the repair and regeneration of cartilage on the surface of knee joints in rats.

In this experiment, the rat knee joint pathological tissue was not collected at the 2nd, 3rd, and 4th weeks after the rat knee joint osteoarthritis modeling. Some scholars believe that the rat knee joint osteoarthritis model has not been completely established at these time points.^30^ Therefore, the pathological collection of rat knee joints was not performed at these time points in this experiment.

## Conclusion

In this study, we have preliminarily explored the correlation between melatonin and OA, the correlation between melatonin and IL‐1β, IL‐6 and COX‐2, and clarified the mechanism of the effect of melatonin on OA. However, there are some shortcomings in this experiment. The correlation between melatonin and OA at the molecular level or even at the genetic level still needs to be further explored to provide a basis for clinical prevention and treatment of OA.

## Author contributions

CHK and HYL conceived the methods of the study, performed the database search, the article selection and data extraction processes, performed the statistical analysis and drafted the manuscript. DY and JW conceived the methods of the study, performed the database search, the article selection and data extraction processes. HY and HWZ conceived the methods of the study and analyzed data. JX and LW helped to perform data extraction processes and draft the manuscript. All authors reviewed the final manuscript. All authors agree to be accountable for all aspects of the work.
